# Human Exposure to Tickborne Relapsing Fever Spirochete *Borrelia* *miyamotoi*, the Netherlands

**DOI:** 10.3201/eid2007.131525

**Published:** 2014-07

**Authors:** Manoj Fonville, Ingrid.H.M. Friesema, Paul D. Hengeveld, Arieke Docters van Leeuwen, Seta Jahfari, Margriet G. Harms, Arnold J.H. van Vliet, Agnetha Hofhuis, Wilfrid van Pelt, Hein Sprong, Cees C. van den Wijngaard

**Affiliations:** National Institute of Public Health and the Environment, Bilthoven, the Netherlands (M. Fonville, I.H.M. Friesema, P.D. Hengeveld, A. Docters van Leeuwen, S. Jahfari, M.G. Harms, A. Hofhuis, W. van Pelt, H. Sprong, C.C. van den Wijngaard);; Wageningen University, Wageningen, the Netherlands, (A.J.H. van Vliet)

**Keywords:** Tickborne diseases, zoonoses, relapsing fever, Lyme disease, Borrelia, borreliosis, miyamotoi, B. burgdorferi sensu lato, the Netherlands

**To the Editor:** *Borrelia* *miyamotoi* is a relatively novel tickborne relapsing fever spirochete, and is a different species than *B. burgdorferi* sensu lato, the causative pathogen of Lyme borreliosis (*1*). *B. miyamotoi* was first isolated in 1995 from *Ixodes persulcatus* ticks in Japan, after which it was detected in ticks in North America, Europe, and Russia (*1*,*2*). *B*. *miyamotoi* infections among humans were first reported in Russia in 2011 (*3*), and in 2013 in the United States (*4*). Recently, the first patient infected with *B. miyamotoi* was reported in the Netherlands (*5*). Conditions reported to be associated with *B*. *miyamotoi* infection were systemic, including malaise and fever, meningoencephalitis, and neurologic symptoms. Because of the nature of these manifestations and because regular diagnostic tests for *B. burgdorferi* will most probably not detect *B*. *miyamotoi* infections (*3*,*5*), *B*. *miyamotoi* infections may remain undiagnosed. Nevertheless, the relationship between *B*. *miyamotoi* infection and illness is not very well established; the case-patients reported, including the patient in the Netherlands, were usually hospitalized, severely ill, and often immunocompromised (*3*–*5*). The extent to which *B*. *miyamotoi* causes infection and disease in immunocompetent persons is unknown. As a first step to indicate the population at risk for infection, we investigated human exposure to *B*. *miyamotoi* in the Netherlands.

To do this, we assessed the *B*. *miyamotoi* infection rate of ticks that had bitten humans. Earlier studies included ticks collected through flagging an area (*1*,*2*); our study provides specific information about the infection rate of ticks feeding on humans. The ticks were collected from persons who reported their tick bites on the website http://www.tekenradar.nl. After removal of the ticks from the skin, the ticks were submitted to the National Institute of Public Health and the Environment. For 1,040 ticks gathered during April–June 2012, we determined tick species, stage of development, and gender by microscopic examination.

We defined the degree of engorgement in 4 categories from unengorged (score 0) to fully engorged (score 3), as visually determined. To isolate DNA, we boiled the ticks with engorgement scores of 0­–1 in ammonium hydroxide (*6*); for ticks with engorgement scores of 2–3, we used the QIAGEN (Valencia, CA . USA) blood and tissue DNA-extraction kit (*7*). We used a *B*. *miyamotoi*–specific real-time PCR based on the flagellin gene for detection of the bacteria (*5*). Quantitative PCR-positive tick lysates were tested with a conventional PCR, which amplifies a fragment of glycerophosphodiester phosphodiesterase (*glpQ*) gene, to confirm the outcome (*5*). These PCR products were sequenced and were identical to *B. miyamotoi* sequences filed in GenBank (AB824855). We determined the presence of *B*. *burgdorferi* DNA with a duplex quantitative PCR using fragments of the outer membrane protein A gene and the flagellin B gene as targets (*7*).

All 1,040 ticks were identified as *Ixodes ricinus*, the most common tick that transmits *B. burgdorferi* in northern Europe (*8*). We detected *B*. *miyamotoi* DNA in 37 ticks (3.6%) using real-time PCR targeting the flagellin gene, which was confirmed for 32 ticks (3.1%) in the conventional PCR targeting the *glpQ* gene. (Technical Appendix Table). In 9 of the 37 ticks positive for *B*. *miyamotoi*, *B*. *burgdorferi* was also detected. Similar to *B. burgdorferi*, the risk of transmission of *B. miyamotoi* is likely to become higher if ticks become engorged with blood; 23 of the 37 (62.2%) *B.* *miyamotoi*–infected ticks were somewhat engorged (score 1–3) and thus had such an increased risk for transmission. All *glpQ* sequences of the detected *B*. *miyamotoi* isolates were identical to the sequence detected in the sample from the patient reported in the Netherlands this year by Hovius et al. (*5*). *B. burgdorferi* DNA was detected in 190 ticks (18.3%) compared with 11.8% detected in a study that included ticks collected through flagging (*9*).

Figure shows that ticks included in the study were submitted from all parts of the country; *B. miyamotoi*– and *B. burgdorferi*–positive ticks were found in almost every region. Of the ≈1 million tick bites per year in the Netherlands (*10*), an estimated 36,000 were by ticks that were infected with *B. miyamotoi,* and 183,000 were by ticks infected with *B. burgdorferi*. This substantial human exposure to *B. miyamotoi* and the reported cases in Russia, the United States, and, recently, the Netherlands (*3*–*5*) raises the question to what extent exposure to *B. miyamotoi* leads to human disease in the general population? These results call for the development of sensitive and specific serologic and molecular tests for *B. miyamotoi* to identify possible patients, which will lead to a better understanding of the clinical spectrum of *B. miyamotoi*–induced disease.

**Figure:**
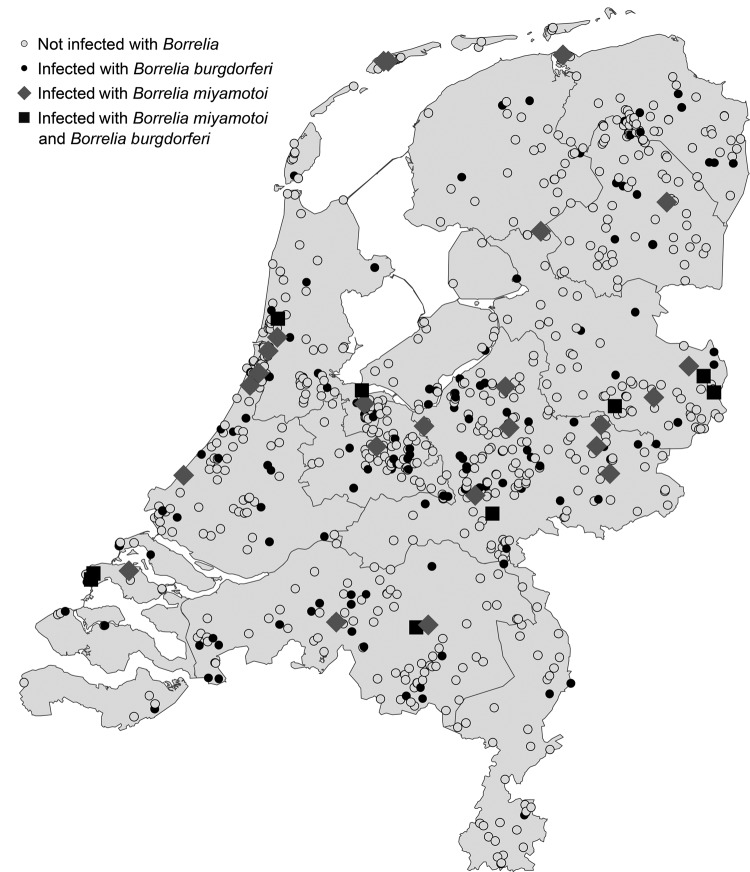
Locations of ticks collected through the website http://www.tekenradar.nl in the Netherlands during summer 2012,. Ticks included in the study were submitted from all parts of the country; ticks positive for *Borrelia* *miyamotoi* and *B*. *burgdorferi* were found in almost every region.

Technical Appendix Infection rates of *Ixodes ricinus* ticks with *Borrelia miyamotoi* and *B*. *burgdorferi*
