# Supplementary material for: Human Exposure to Tickborne Relapsing Fever Spirochete Borrelia miyamotoi, the Netherlands
Source: Emerg Infect Dis. 2014 Jul;20(7):1244–6. doi: 10.3201/eid2007.131525 (PMC4073841; doi:10.3201/eid2007.131525)
Supplement: Technical Appendix — Infection rates of Ixodes ricinus ticks with Borrelia miyamotoi and B. burgdorferi [file 13-1525-Techapp-s1.pdf]

Article DOI: 10.3201/eid2007.131525

Human Exposure to Tickborne Relapsing Fever Spirochete *Borrelia miyamotoi*, the Netherlands

Online Technical Appendix Table. Tick infection rates by *B. miyamotoi* and *B. burgdorferi* in 1,040 *Ixodes ricinus* ticks, by developmental stage and degree of engorgement*

Stage engorgement	No.tested (%)	DNA detected for: *B. burgdorferi* (%)	*B. miyamotoi* (%)
Larval	29	0	2 (6.9)
Score 0	10 (34.5)	0	1 (10.0)
Score 1-3	19 (65.5)	0	1 (5.3)
Nymphal	792	122 (15.4)	24 (3.0)
Score 0	310 (39.1)	51 (16.5)	8 (2.6)
Score 1-3	482 (60.9)	71 (14.7)	16 (3.3)
Adult	219	68 (31.1)	11 (5.0)
Score 0	136 (62.1)	50 (36.8)	5 (3.7)
Score 1-3	83 (37.9)	18 (21.7)	6 (7.2)
Total	1,040	190 (18.3)	37 (3.6)
Score 0	456 (43.8)	101 (22.1)	14 (3.1)
Score 1-3	584 (56.2)	89 (15.2)	23 (3.9)

*Microscopic examination consisted of determining tick species, stage of development and gender using standard keys (1). Ticks were scored as unengorged (score 0) versus any degree of engorgement (score 1–3).

Reference

1. Estrada-Peña A. Ticks of domestic animals in the Mediterranean Region, a guide to identification of species. Zaragoza (Spain): University of Zaragoza; 2004.
